# A cluster randomised trial to assess the impact of clinical pathways on AMI management in rural Australian emergency departments

**DOI:** 10.1186/1472-6963-9-83

**Published:** 2009-05-25

**Authors:** Leigh D Kinsman, Penny Buykx, John S Humphreys, Pamela C Snow, Jon Willis

**Affiliations:** 1 School of Rural Health, Monash University, PO Box 666, Bendigo, VIC 3552, Australia; 2 School of Psychology, Psychiatry, and Psychological Medicine, Monash University, PO Box 666, Bendigo, VIC 3552, Australia; 3 School of Public Health, La Trobe University, PO Box 199, Bendigo, VIC 3552, Australia

## Abstract

**Background:**

People living in rural Australia are more likely to die in hospital following an acute myocardial infarction than those living in major cities. While several factors, including time taken to access hospital care, contribute to this risk, it is also partially attributable to the lower uptake of evidence-based guidelines for the administration of thrombolytic drugs in rural emergency departments where up to one-third of eligible patients do not receive this life-saving intervention. Clinical pathways have the potential to link evidence to practice by integrating guidelines into local systems, but their impact has been hampered by variable implementation strategies and sub-optimal research designs. The purpose of this study is to determine the impact of a five-step clinical pathways implementation process on the timely and efficient administration of thrombolytic drugs for acute myocardial infarctions managed in rural Australian emergency departments.

**Methods/Design:**

The design is a two-arm, cluster-randomised trial with rural hospital emergency departments that treat and do not routinely transfer acute myocardial infarction patients. Six rural hospitals in the state of Victoria will participate, with three in the intervention group and three in the control group. Intervention hospitals will participate in a five-step clinical pathway implementation process: engagement of clinicians, pathway development according to local resources and systems, reminders, education, and audit and feedback. Hospitals in the control group will each receive a hard copy of Australian national guidelines for chest pain and acute myocardial infarction management. Each group will include 90 cases to give a power of 80% at 5% significance level for the two primary outcome measures: proportion of those eligible for thrombolysis receiving the drug and time to delivery of thrombolytic drug.

**Discussion:**

Improved compliance with thrombolytic guidelines via clinical pathways will increase acute myocardial infarction survival rates in rural hospitals and thereby help to reduce rural-urban mortality inequalities. Such knowledge translation has the potential to be adapted for a range of clinical problems in a wide array of settings.

**Trial registration:**

Australia New Zealand Clinical Trials Registry code ACTRN12608000209392.

## Background

Coronary heart disease is a major health problem in Australia with a reported 637,900 people diagnosed in 2004–05[1]. Acute myocardial infarction (AMI), a manifestation of coronary heart disease, is the leading cause of sudden death in the Australian population accounting for one in ten deaths[1,2]

Timely and efficient delivery of thrombolytic drugs to individuals suffering an acute myocardial infarction significantly improves mortality and morbidity and is a major aim of early treatment when invasive coronary reperfusion services are not readily available[3,4]. The National Heart Foundation of Australia (NHFA) and the Cardiac Society of Australia and New Zealand (CSANZ) jointly produced clinical guidelines for the management of AMI, including the administration of thrombolytic drugs when percutaneous coronary intervention services are unavailable. These guidelines provide precise recommendations directing which patients should receive a thrombolytic drug based on presenting symptoms, electrocardiographic (ECG) findings and contraindications[5].

People living in rural Australia are more likely to die in hospital following an AMI than people in major cities[6]. Whilst there are obvious problems associated with distances people travel to access treatment, there also appears to be lower uptake of national guidelines for thrombolytic drugs in rural areas, where up to one-third of eligible patients do not receive this life-saving intervention[7]. Peak Australian bodies, including the NHFA, the Australasian College of Emergency Medicine (ACEM) and the Cardiac Society of Australia and New Zealand (CSANZ) have promoted the development of local systems to improve to uptake of national guidelines [8].

Clinical pathways are structured, locally developed multidisciplinary care plans for specific clinical problems that take into account local resources and make explicit the local system for clinicians. They are an important tool for linking evidence to practice and can enhance adherence to guidelines[9]. However, sub-optimal implementation processes and study design have compromised the quality of current evidence regarding the impact of clinical pathways on practice[10].

Active, multifaceted approaches have been shown to improve adherence to guidelines in clinical settings over passive dissemination strategies. Active approaches include a combination of audit and feedback, reminder systems, engaging clinicians and targeted education[11,12].

The aim of this cluster randomised controlled trial is to measure the impact of a multifaceted process of clinical pathways implementation on thrombolytic delivery for AMIs managed in rural emergency departments in Australia.

## Methods

The "Acute myocardial infarction: investigating evidence-based practice to address the rural disadvantage" study is being coordinated by the Monash University School of Rural Health with rural hospitals in the state of Victoria. In this setting, all hospitals outside of major cities are categorised as rural [1]. The study is funded by a Faculty Strategic Grant from the Faculty of Medicine, Nursing and Health Sciences at Monash University and was registered with the Australia New Zealand Clinical Trials Registry (code: 12608000209392) in April 2008.

Ethics approval has been obtained from the Monash University Standing Committee on Ethics involving Humans and relevant hospital human research ethics committees. Individual informed consent is not feasible within this research design and the decision to undertake the research is the responsibility of participating institutions.

### Aim

To determine if a five-step implementation process of clinical pathways for AMI improves the proportion of patients receiving a thrombolytic drug and reduces the average time from presentation at hospital to thrombolytic administration in rural emergency departments.

### Objectives

1. To assess performance of rural hospitals against NHFA recommendations for administration of thrombolytic drugs for AMI; and

2. To measure the effect of a five step intervention for the implementation of clinical pathways in rural hospitals for AMI on:

a) the proportion of eligible patients receiving a thrombolytic drug, and

b) the time from presentation at hospital to administration of a thrombolytic drug.

### Study Design

A randomised controlled trial will be conducted involving six rural hospitals that treat and do not routinely transfer AMI patients. As displayed in table 1, pairs of hospitals were matched according to the anticipated number of eligible patients and on the basis of geographical separation in order to minimise the risk of a control hospital being influenced by procedures at an intervention hospital. Randomisation within pairs to either the intervention (n = 3) or control (n = 3) groups occurred by a simple coin toss.

**Table 1:** Geographic characteristics and anticipated number of patients per site.

	**Kilometres between matched hospitals^a^**	**Mean number of eligible patients per year^b^**
Intervention Control	318	60 55

Intervention Control	463	50 50

Intervention Control	155	15 20

^a ^Source: Google Earth

^b ^Estimates based on previous research [7]

### Sample size

A 20 minute reduction in time to thrombolytic administration represents a modest improvement[7]. A total post intervention sample size of 90 patients is required to detect a 20 minute reduction in thrombolytic delivery time and a 10% improvement in eligible patients receiving thrombolysis (significance level of 5%, power of 0.8, and intra-cluster correlation coefficient of 0.283)[13]. Based on anticipated total attendance rates (i.e. 125 eligible case per annum in each condition) there will be nine months of pre and nine months of post intervention data collected.

### Intervention

The three intervention hospitals will participate in the five step implementation process described below. The control hospitals will receive a hard copy of the relevant NHFA guidelines. The evidence-based five step intervention within emergency departments to implement clinical pathways will be a combination of processes proposed by Doherty & Jones[14] and Kinsman et al.[7]. In brief, the three month implementation process entails:

#### 1. Engaging clinicians

The Chief Investigator will hold group discussions with emergency department medical and nursing staff regarding barriers and facilitators for clinical pathways whilst local clinicians will be recruited as Research Assistants. It is hoped that the employment of local clinicians will facilitate local opinion leadership.

#### 2. Clinical pathway development

Hospital-specific clinical pathways will be developed by Research Assistants in collaboration with clinicians at each emergency department. Pathway content will be consistent with NHFA and CSANZ guidelines for chest pain and AMI management, and will incorporate local resources and systems.

#### 3. Reminders

Reminder visits by the Chief Investigator will occur twice following implementation to liaise with clinical staff. Research Assistants will be tasked with reminding medical and nursing staff about the clinical pathway to promote utilisation.

#### 4. Education

The Research Assistant and Chief Investigator will facilitate education sessions during implementation to review evidence underpinning the clinical pathway and reinforce the role of the pathway itself. All staff will receive written material regarding thrombolytic drugs, including summaries of the national guidelines.

#### 5. Audit and feedback

Audit results reflecting compliance with the clinical pathway and thrombolysis administration will be communicated by the Chief Investigator to emergency department staff twice during the implementation of the clinical pathway. Participants will be encouraged to express their views on the advantages and pit-falls of the clinical pathway.

### Data Collection

Medical records will be identified by an International Classification of Diseases (ICD 10) report and audited using a specifically-designed data protocol. Initial data will include type of infarct, gender and age. Information regarding whether criteria were met for a thrombolytic drug will be checked. If criteria were met, then whether a thrombolytic was administered will be recorded, as will the time in minutes from presentation to administration. The level of adoption of the clinical pathway will be measured by identifying the presence of the pathway in audited records and by degree of documented completion of the pathway. A co-investigator will review 10% of the medical records to confirm accuracy of data extraction.

### Data Analysis

Use of thrombolytic drugs will be categorised as yes/no for each eligible patient, and intervention and control hospital groups will be compared at baseline and follow-up using a standard Chi-squared test. An independent sample *t*-test will be used to compare time to thrombolytic delivery between intervention and control groups at baseline and follow-up. Matched *t*-tests will assess differences between baseline and follow-up for both the intervention and control groups. Between-group comparisons allowing for the clustering of data will be undertaken. Documented usage of the clinical pathway will be measured by simple descriptive statistics such as percentage of AMI cases in which the pathway was used and proportion of the chart completed.

## Discussion

The evidence for clinical pathways remains inconclusive and the reasons for the variable results reported may relate to both implementation strategies and research designs. It is important to determine rigorously whether clinical pathways have an integral role to play in enhancing compliance with evidence-based practice. Randomisation of individual patients within a single ward or hospital to complex interventions such as clinical pathways will lead to contamination of comparable samples and a high risk of bias. In these situations a cluster randomised controlled trial is a preferred method to minimise the influence an intervention may have on clinical practice in control groups.

This study will contribute to the knowledge base on what is required to translate evidence into practice in rural settings. In this study improved compliance with thrombolytic guidelines via clinical pathways will increase AMI survival rates in rural hospitals and help reduce rural-urban mortality inequalities. Such translation science will have the potential to be applied in a range of clinical settings for many different conditions.

## Competing interests

The authors declare that they have no competing interests.

## Authors' contributions

LK, JH, PS and JW were involved in study design and writing the manuscript. PB recruited rural hospitals into the study and was involved in writing the manuscript. All authors read and approved the final manuscript.

## Pre-publication history

The pre-publication history for this paper can be accessed here:


